# Chronic effects of temperature on mortality in the Southeastern USA using satellite-based exposure metrics

**DOI:** 10.1038/srep30161

**Published:** 2016-07-20

**Authors:** Liuhua Shi, Pengfei Liu, Yan Wang, Antonella Zanobetti, Anna Kosheleva, Petros Koutrakis, Joel Schwartz

**Affiliations:** 1 Harvard T.H. Chan School of Public Health, Department of Environmental Health, Harvard University, Boston, Massachusetts, USA; 2 Harvard John A. Paulson School of Engineering and Applied Sciences, Harvard University, Cambridge, Massachusetts, USA; 3 Harvard T.H. Chan School of Public Health, Department of Epidemiology, Harvard University, Boston, Massachusetts, USA

## Abstract

Climate change may affect human health, particularly for elderly individuals who are vulnerable to temperature changes. While many studies have investigated the acute effects of heat, only a few have dealt with the chronic ones. We have examined the effects of seasonal temperatures on survival of the elderly in the Southeastern USA, where a large fraction of subpopulation resides. We found that both seasonal mean temperature and its standard deviation (SD) affected long-term survival among the 13 million Medicare beneficiaries (aged 65+) in this region during 2000–2013. A 1 °C increase in summer mean temperature corresponded to an increase of 2.5% in death rate. Whereas, 1 °C increase in winter mean temperature was associated with a decrease of 1.5%. Increases in seasonal temperature SD also influence mortality. We decomposed seasonal mean temperature and its temperature SD into long-term geographic contrasts between ZIP codes and annual anomalies within ZIP code. Effect modifications by different subgroups were also examined to find out whether certain individuals are more vulnerable. Our findings will be critical to future efforts assessing health risks related to the future climate change.

Climate change affects human health in many ways, including the effects of severe weather, hurricanes, flooding, winter storms, drought, etc. One manifestation of climate change over the past few decades is global warming due to greenhouse gases emissions^1^. The Third National Climate Assessment (NCA3) stated that the last decade was America’s warmest on record and temperature rise in the country is expected to continue^2^. This change poses a serious threat to human health. A review study reported that global warming has already claimed over 150,000 lives per year over the past few decades worldwide^3^. The link between ambient air temperature (*T*_*a*_) and human health in different climate zones is also of great interest to researchers^4^,^5^,^6^,^7^,^8^,^9^,^10^.

Numerous time-series studies and case-crossover studies have shown that short-term exposure to several days of very hot or cold weather is associated with increased mortality^11^,^12^,^13^,^14^,^15^,^16^,^17^,^18^. However, for long-term temperature change, e.g. yearly, the impact on mortality is not yet well understood. Recently, we examined the effect of long-term temperature change on mortality, and found that the impact of temperature over a full season may differ from the short-term effects^19^,^20^. This is because it estimates effects net of short-term harvesting, and inclusive of cumulative effects of long-term exposures. In the current study, we focus on associations of annual human mortality and seasonal temperature patterns, to better quantify the longer-term effects of changing temperature patterns.

In addition to seasonal mean temperatures, the potential mortality risk from within-season temperature variability has been recognized recently^19^,^20^,^21^,^22^. A corollary to individuals adapting to their usual temperatures is that the greater deviation from normal conditions, the larger the impact^23^,^24^,^25^. In the present work, we investigated the effects of both mean temperature and its variability during the summer and winter seasons.

Previous temperature-related health studies are primarily limited to surface air temperature (*T*_*a*_) data obtained at sparsely located weather stations. The lack of spatially- and temporally-resolved data introduces an exposure measurement error. This may contain not only Berkson error but also classical error and lead to biased health effect estimates^26^. In addition, it fails to capture spatial variability in temperature due to urban heat islands, green space, etc. Based on the high resolution surface temperature (*T*_*s*_) derived from satellite measurements, a statistical modeling approach was presented to estimate daily *T*_*a*_ at fine geographic scale (1 km × 1 km) for the northeastern USA^27^. This dataset has been used in several epidemiological studies to assess health effects associated with temperature conditions. For example, Kloog *et al*. (2015) investigated the association between air temperature and birth outcomes in Massachusetts^28^. In Shi *et al*. (2015) we have studied the effects of spatial contrasts and annual anomalies of local *T*_*a*_ for the all-cause mortality of Medicare population (aged 65+) in New England^20^.

Using a similar modeling framework, we recently retrieved the high-resolution *T*_*a*_ dataset for the Southeastern USA (SEUS) for the period 2000–2014, as we describe in Shi *et al*.^29^ This made possible to investigate the health effects for elderly in this area. The temperature-induced health effects are of particular interest for the SEUS because a large fraction of older Americans has moved to this area, in part due to the belief that the warm and mild climate is beneficial for their health. This population, however, is potentially most vulnerable to temperature changes, and while mild winters may protect them from cold effects, they may be more vulnerable to heat. Quantitative knowledge about the elderly’ responses to climate change for this particular region is clearly needed in the era of global warming.

Herein, the aim was to investigate the chronic health effects of seasonal mean temperature and its variability on annual mortality for elderly in the SEUS. Toward this end, we analyzed data from the Medicare cohort (aged 65+, 13 million) during the period 2000 to 2013. By temperature variability we mean the standard deviation (SD) of within-season daily mean temperature for the summer months (June, July, and August) and winter months (December, January, and February). To assess the impact of temperature by location and over time, we separately investigated how 14-year average for each ZIP code and annual anomaly (difference between the seasonal mean and the 14-year average for each ZIP code) associate with annual mortality. The former association represents the effects of spatial contrasts in typical local temperature patterns. For example, Miami is warmer than Nashville in winter for many years, and we considered this effect on mortality as the effect of spatial contrasts in temperature. Because it represents the prevailing climate for many years, it may reflect adaptations to such prevailing climate as well. The latter represents the effect of year-to-year variation of the prevailing seasonal temperature patterns. We also tested for effect modification by different subgroup populations to find out whether certain subgroups are more vulnerable than others. Importantly, our analysis was on the individual level, rather than aggregating deaths for a population. Finally, we compared the results for SEUS obtained in this work with our previous results for the New England area^20^, which exhibits cold winters and mild summers. Such comparison can reveal the differences in different climate zones.

## Results

### Characteristics of the study population and exposure

We followed 13 million Medicare beneficiaries in SEUS during 2000–2013, with an average of 7.3 years of follow-up. Table 1 shows the study population characteristics. Females and relatively older individuals (aged 75+) accounted for 55.8% and 53.4% of the study population, respectively. Furthermore, 82.5% of the study population was white, followed by black (13.7%) and the other race group (3.8%). During the study period, there were 4.7 million (35.5%) all cause deaths.

**Figure 1:**
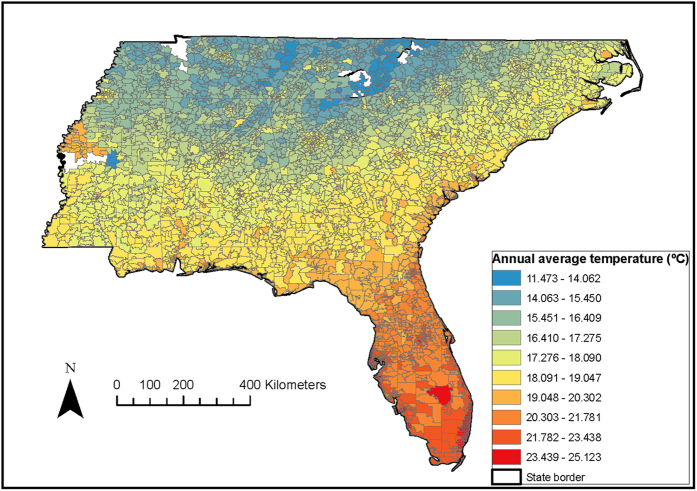
Spatial pattern of estimated temperature (°C), averaged over the 2011 for the Southeastern USA. By using ArcGIS Desktop 10.3 (http://desktop.arcgis.com/en/arcmap/), each ZIP code centroid was linked to the nearest 1 km temperature grid, and then the 1 km daily temperature predictions were assigned to this ZIP code.

Because the deaths are coded at the ZIP code level, the nearest 1 km × 1 km temperature prediction to the centroid of the ZIP code was assigned. Figure 1 presents the distribution of the predicted annual average temperature for a sample year (2011) in 4,450 ZIP codes across the SEUS. We observed higher temperatures for areas closer to the shoreline as compared to those predicted for the inland areas. Table 2 reports the descriptive statistics for seasonal mean temperature and its variability across the SEUS during the summer and winter seasons for the period 2000–2013. The summer and winter mean temperatures were 26 °C and 9.4 °C, respectively.

### Main effects

Table 3 presents a summary of the effects of annual anomalies, spatial contrasts, and the overall effect estimates from the main analyses. The results from the sensitivity analyses are displayed in the Supplementary Table S2. They are expressed as percent increases in mortality for each 1 °C increment of exposure index. In the main analyses, a rise in summer mean temperature of 1 °C was associated with an overall 2.46% (95% CI: 2.33, 2.59%) increase in death rate. This effect was contributed by both annual anomaly (temporal variation) and spatial contrast, indicating that the death rate is higher in areas or years with higher summer temperatures and suggesting little acclimatization to hot. In winter, a 1 °C increase in mean temperature reduced the annual death rate by 1.46% (95% CI: 1.42, 1.50%). The death rate was lower for a warmer winter either temporally or spatially. For all factors related to the seasonal mean temperatures, the health effects are statistically significant (*p* < 0.001).

For increases in temperature SDs in summer and winter, the overall effects were harmful. However the sensitivity analyses showed that these harmful effects of swings in temperatures were mainly attributed to heat waves and cold waves (*cf*. Supplementary Table S2). All analyses observed opposite effects of spatial and temporal variations in temperature SDs. It was annual anomalies of temperature SDs in both summer and winter that drove the increased mortality. For anomalies, a 1 °C increase in temperature SDs yielded 3.71% (95% CI: 3.21, 4.22%) and 0.59% (95% CI: 0.37, 0.81%) increases in annual deaths in summer and winter, respectively. These results suggest a higher risk for years having more variable weather compared to the 14-year average. However, the spatial variation can play a different role. We observed 18.46% (95% CI: 17.67, 19.25%) and 3.49% (95% CI: 3.08, 3.90%) decreases in mortality for a 1 °C increase in temperature SDs spatially in summer and winter, respectively. These results indicate health benefits for those living in ZIP codes with more variable temperatures in both summer and winter. This finding is in contrast with the previous results for New England, where the spatial contrasts of SDs increased the health risks. This comparison could suggest different roles of temperature variability in different climate zones. However, we cannot rule out the possibility that the spatial contrasts in SDs correlate with some spatial confounders that were not included in the model, such as the distance to the coast, which may affect the mortality.

### Effect modification

**Figure 2:**
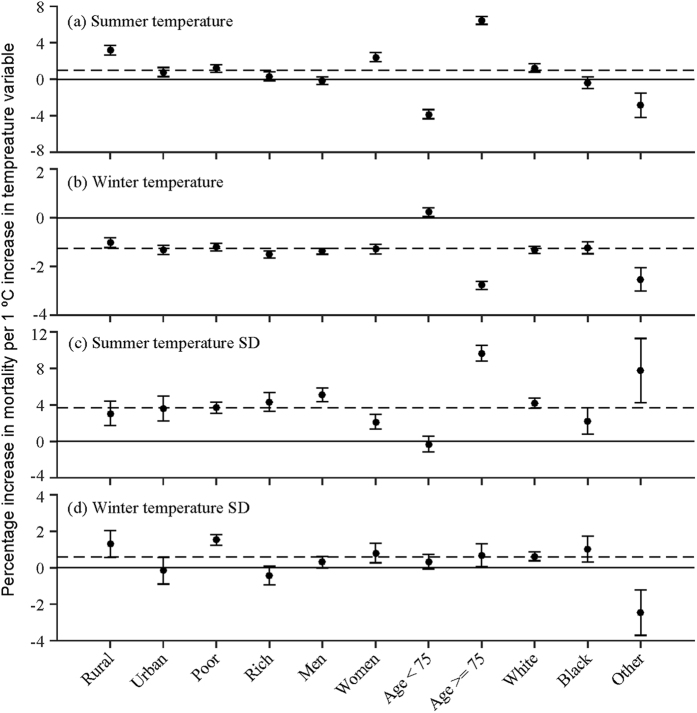
Modifications of the effect for annual anomalies of different temperature indices by socio-economic and individual characteristics. It displays the percent increases of death (95% CI) for each 1 °C increase in (**a**) summer mean temperature annual anomaly (**b**) winter mean temperature annual anomaly (**c**) summer temperature standard deviation annual anomaly (**d**) winter temperature standard deviation annual anomaly in each subgroup, respectively. Red indicates increased risk of death, and blue stands for decreased risk of death. Error bars stand for 95% confidence intervals.

**Figure 3:**
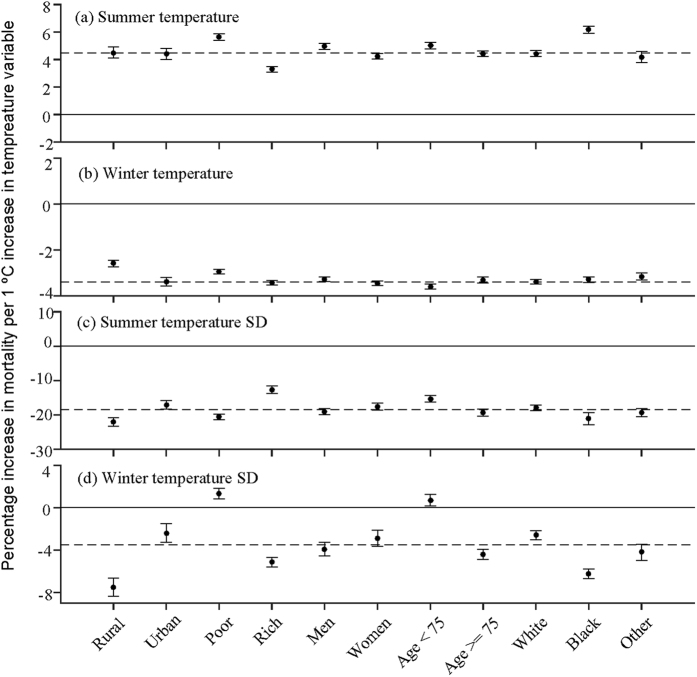
Modifications of the effect for spatial variability in different temperature indices by socio-economic and individual characteristics. It displays the percent increases of death (95% CI) for each 1 °C increase in (**a**) spatial contrasts of summer mean temperature (**b**) spatial contrasts of winter mean temperature (**c**) spatial contrasts of summer temperature standard deviation (**d**) spatial contrasts of winter temperature standard deviation in each subgroup, respectively. Red indicates increased risk of death, and blue stands for decreased risk of death. Error bars stand for 95% confidence intervals.

We tested population density, poverty, sex, age, and race as modifiers of the effects of temperature singularly, to examine whether certain subgroups are more vulnerable. We illustrate the effects of temporal variation and spatial contrast in Figs 2 and 3, respectively. Figure 2 suggests that subjects residing in areas with lower population density are more sensitive to changes in summer mean temperature and winter temperature SD, as opposed to city dwellers. We observed a similar sensitivity for poorer areas, possibly because residents cannot afford air conditioning and heating and have limited access to health care. We also found that city dwellers and affluent subjects benefited more from warmer winters. In addition, the relatively older age group (aged 75+) has been shown to be particularly sensitive to changes in seasonal mean temperature, which is consistent with a recent study^30^. In contrast to men, women were more sensitive to increased summer mean temperature but less to increased seasonal temperature SDs. The cause for these differences, however, is unclear. Furthermore, there are small differences in response of the Black individuals to changes in temperature indices compared to those of White, but the other race group responded differently relative to White individuals. It is likely that the elderly from other race groups emigrated from warm areas, such as Southeast Asia and Mexico, so they may have benefited from adaptation to warmer summers and winters.

For long-term contrasts between ZIP codes, we saw consistent decreases in mortality across population groups for winter mean, summer SD, and winter SD. This indicates health benefits of living in an area with warmer winter, or in an area with more within-season fluctuations (see Fig. 3). Living in an area with warmer summer mean temperature, however, has an adverse health effect (Fig. 3a). Subjects in poorer ZIP codes are at higher risk for warmer summers and more variable winters, and the relatively younger age group (65–74 y) exhibited the similar pattern. Subjects residing in urban areas and the rich benefited more from living in ZIP codes with warmer winters. This is consistent with our study in New England^20^. We found men to be more sensitive to changes in mean summer temperature. We also observed that changes in temperature mean and SD have a larger effect for the Black than the White.

We also fitted models with simultaneous interactions for which we observed a significant difference between urban and rural. By simultaneous interaction, we mean including five interaction terms between individual-level (age, sex, and race) and area-level factors (dummy variables for urban/rural, poor/rich) with those exposure indices in the models simultaneously. Most of the effect sizes shrank, but the directions of interactions remained the same and the differences between urban and rural were still significant with *p*-values < 0.05 (results were not shown).

## Discussion

This study examined how the elderly in the SEUS responded to the spatial and temporal changes in temperature during the last decade using local *T*_*a*_ predictions. Unlike previous time series studies of daily temperature and aggregate daily deaths in a city, we have followed individuals, controlled for individual risk factors, estimated the annual risk of death for each person and follow-up year, and used local estimates of temperature in the ZIP code of origin instead of data of sparsely distributed weather stations. This enabled us to estimate effects on annual death rates instead of daily death rates, to use more precise exposure estimates, and to capture the effects of spatial variation in temperature. Our findings suggest that even in a region approaching universal air conditioning prevalence, warmer summer temperatures result in higher death rates. Moreover, advances in deaths (harvesting) by only a few weeks or months due to summer weather would not bias annual mortality rates, therefore indicating the life shortening of these exposures is not trivial. Furthermore, ZIP codes with higher long-term average summer temperatures had higher mortality rates. This suggests that in areas with hot summers like the SEUS (summer mean temperature = 26 °C) adaptation has ran its course, and elevated long-term temperatures are associated with greater death rates. We observed higher death rates for years with warmer summers. Additionally, air conditioning prevalence in this region is almost saturated, and there may be little room for further adaptation. Hence, such locations may be particularly sensitive to rising summer temperatures in the future.

We found both similarities and differences between our SEUS and New England studies. Increasing summer mean temperature is associated with increased mortality, and increasing winter mean temperature has the expected effect of lowering mortality in both. For both regions, increased variability in summertime and wintertime temperatures raises the risk of death. However, increases in temperature variability in New England can have similar or even larger estimated effects on mortality than increases in mean temperature, while in the SEUS the effects of mean temperature are higher. A possible explanation is that the effects of seasonal temperature SDs may depend on the mean temperature. That is, a larger SD in warm areas may mean differently from a larger SD in cold areas. For example, the SEUS is considerably warmer than the New England, with respective summer mean temperatures of 26 °C and 16 °C and respective winter mean temperatures of 9 °C and 2 °C during the study period. Therefore, a larger SD in the SEUS may mean more breaks in hot weather, while in the New England, a larger SD may mean more episodes of hot weather in summer and more episodes of cold weather in winter. However, this hypothesis needs to be tested in future studies for other climate zones.

In the univariate interaction analyses, we found that subjects living in less densely populated areas were at higher risk of death. One possible explanation is that some population characteristics that were not controlled in the model can be different in urban and rural areas. In addition, access to health services, public health prevention measure outreach, and warning system dissemination can explain some of the difference observed between urban and rural. We also adjusted for four more interactions simultaneously to test the robustness of the dummy variable for urban and rural. This analysis showed that by including simultaneous interactions, the difference between urban and rural decreased, but none of the risk estimates changed sign or lost significance. This suggests that the differences we observed between urban and rural were robust.

One major feature of this study is that it is a population based Medicare cohort, rather than a convenience sample. It also allows us to examine populations living distant from a NOAA temperature monitor. Therefore we are studying the entire population aged 65 and over in the SEUS. This allowed us to identify persons living in rural areas as at higher risk.

One limitation of the present study is that the Medicare cohort does not provide information on several potential risk factors for mortality, such as smoking, diet, etc. Hence we cannot exclude the possibility of confounding from these risk factors. We believe it is unlikely that these factors would be associated with temperature (except for possible time trends, which are removed in our study), but it remains a limitation. Another limitation is that Medicare does not provide the underlying cause of death, which is necessary to study susceptibility and understand possible pathways. Thirdly, although the Medicare cohort includes most of the elderly aged 65+ (around 70%), and not those who did not retire or receive health insurance covered by their jobs, potential biases might occur when we generalize these conclusions to the entire elderly population.

As a caveat, the health effects of climate change reported in the present study considered only the pathway via temperature changes. The health effect of other pathways, such as winter storms, flooding, and drought should be investigated in future studies.

In conclusion, this study adds more evidence of the chronic effect of temperature on mortality. Further, it suggests that even in the Southeastern USA, a region with considerable adaption to hot summers and a very high prevalence of air conditioning, ZIP codes with higher summer temperature or temperature SD had higher death rates. This suggests that temperature related mortality is already present at non-trivial levels, and adaptation ability is limited. Risk coefficients in this study can be used to quantify human health impacts for future assessments of climate. These efforts aim to inform public health officials, decision makers, and scientists to better understand health risks.

## Methods

### Study domain

The spatial domain of this study includes the Southeastern USA (SEUS), comprising the states of Florida, Georgia, North Carolina, South Carolina, Mississippi, Alabama and Tennessee (*cf* Fig. 1).

### Study population

The study population comprised fee-for-service Medicare beneficiaries, who were aged 65 and older, for the years 2000–2013 in the study domain. Individual mortality records were obtained from the US Medicare data. For each subject, the Medicare beneficiary denominator file contains information on enrollment in Medicare, the date of death, and information on age, sex, race, and ZIP code of residence. The Medicare Provider Analysis and Review (MEDPAR) file provides records for each hospital admission with primary and secondary diagnoses, and the number of coronary care and medical intensive care days for each of the Medicare beneficiaries. Based on the International Classification of Diseases (ICD) codes, we defined four categorical variables for the Medicare beneficiaries on whether they had ever been admitted due to chronic obstructive pulmonary disease (COPD, ICD-9: 491, 492, 496), congestive heart failure (CHF, ICD-9: 428), myocardial infarction (MI, ICD-9: 410), and diabetes (ICD-9: 250), respectively.

Subjects alive on January 1^st^ of the year following the year in which they joined Medicare were entered into the open cohort for survival, and follow-up periods (our time metric) were calendar years. Using their individual level data, we constructed a counting process survival dataset with Andersen Gill formation^31^ for the study population.

### Exposure data

Recently, Kloog *et al*. (2014) presented a statistical modeling approach and assessed daily mean *T*_*a*_ in the Northeastern USA using satellite-derived surface temperature (*T*_*s*_) measurements. A similar approach was applied for estimating daily mean ambient temperature in the SEUS at a spatial resolution of 1 km × 1 km for years 2000–2013. The present study employed these high-resolution *T*_*a*_ exposure estimates.

Specifically, we calibrated the *T*_*s*_−*T*_*a*_ relationship on *each day* using mixed linear models, which allowed us to predict daily *T*_*a*_ in grid cells with available *T*_*s*_ measurements in the study domain. The retrieved *T*_*a*_ was cross validated using monitoring data from weather stations, and a high out-of-sample *R*^2^ of 0.952 indicated excellent model performance. To impute *T*_*a*_ data for grid cells or days when *T*_*s*_ measurements were unavailable, linear regression on nearby monitors and spatio-temporal smoothing was used. A good agreement was achieved when comparing the daily *T*_*a*_ estimations to reanalysis datasets. More details are described elsewhere^29^.

Because the deaths were coded at the ZIP code level, the 1 km grid-cell temperature exposures were matched to ZIP codes by linking the ZIP code centroid to the nearest temperature grid. Using the daily mean summertime (June-August) and wintertime (December-February) temperature, we calculated for each ZIP code and each year the seasonal mean temperature and temperature standard deviations for summer and winter, respectively.

### Covariates

Medicare provides individual level information as mentioned above. ZIP code tabulation area level socio-economic variables were obtained from the U.S. Census Bureau 2000 Census Summary File 3, including population density, percent of less educated, percent below the poverty level, and percent of median value for owner occupied housing units. In addition, county-level percent of age-adjusted diabetes and percent of lack of physical activity were obtained from the CDC Behavioral Risk Factor Surveillance survey.

### Statistical methods

We analyzed the survival data with Andersen Gill formulation by using extended Cox’s proportional hazard models (Proc PHREG, SAS). For each subject survival times were 1 year periods from entering the cohort until death (failure) or December 2013 (censoring). The four exposure metrics included: summer mean temperature, winter mean temperature, summer temperature SD and winter temperature SD in each follow-up year. We entered these four variables into the models simultaneously, to separate their independent associations with mortality. This means that when we looked at the effect of one exposure index, all other exposure indices were held constant, and the auto-correlation between these indices would not be an issue. The independent associations with mortality for these four variables were referred to as the overall effect for each exposure index.

In addition to the overall effect, we also investigated the separate effects of year-to-year variations and place-to-place variations in exposure indices. Specifically, we fit a model with two terms for each temperature index, to capture both the spatial variation and temporal variation, respectively. Each index was defined as its long-term average in that ZIP code over the study period, plus the difference (anomaly) from the long-term average for this ZIP code in a follow-up year. With all eight terms entered into the model simultaneously, the long-term average for each ZIP code captures purely geographic contrasts, and the anomaly indicates the effects of year-to-year variations within each ZIP code.

In all models we controlled for individual risk factors, such as age, sex, race, number of days spent in coronary care unit or medical intensive care unit, and any previous admission of COPD, CHF, MI, and diabetes. We also adjusted for dummy variables for year of follow-up, such that the effect estimates could not be confounded by variables that varied across years (avoid confounding by long-term time trends). Additionally, ZIP code level percent of less educated, percent below the poverty level, population density, percent of median value for owner occupied housing units, as well as county-level percentage of age-adjusted diabetes and percentage of lack of physical activity were adjusted in all models. The correlation matrix for these risk factors can be found in Supplementary Table S1. These models stratified on age groups (5 year categories), sex, and race (white, black, and others), allowing for possible non-proportionality of the survival rates.

We performed some sensitivity analyses, by restricting the cohort to 8 years from 2000–2007, and by controlling for the number of heat waves and cold waves, respectively. We defined heat wave and cold wave as three consecutive days at or above the 95^th^ percentile temperature and at or below the 5^th^ percentile temperature in that ZIP code, respectively. Then we counted how many heat waves and cold waves occurred in each summer and winter respectively for each ZIP code.

We also examined the univariate effect modifications by age (65–75 vs 76 and over), sex, and race (white, black, and other), poverty (less than the mean owner-occupied housing units, poor, and otherwise rich) and population density (less than 33^rd^ percentile, rural, and otherwise urban), by adding interaction terms between such variables and each of the eight temperature variables in the model. By univariate effect modification, we mean each model only included one interaction term between a covariate and a temperature variable, with all other covariates controlled in the same way as the main analyses. We also fitted models with simultaneous interactions of sex, age (65–75 vs 76 and over), race, a dummy for rural/urban, and a dummy for poor/rich, to examine the robustness of these SES variables. By simultaneous interactions, we mean a model included several interaction terms between the covariates and a temperature variable simultaneously.

## Additional Information

**How to cite this article**: Shi, L. *et al*. Chronic effects of temperature on mortality in the Southeastern USA using satellite-based exposure metrics. *Sci. Rep.* **6**, 30161; doi: 10.1038/srep30161 (2016).

## Supplementary Material

Supplementary Information

## Figures and Tables

**Table 1:** Characteristics of the study population across the Southeastern USA for the years 2000–2013.

Characteristics	N	%
Total Population	13,369,590	100
Deaths	4,740,247	35.46
Sex		
** **Male	5,903,823	44.16
** **Female	7,465,767	55.84
Race		
** **White	11,025,850	82.47
** **Black	1,835,599	13.73
** **Other	508,141	3.80
Age		
** **65–74	6,233,796	46.63
** **> = 75	7,135,794	53.37

**Table 2:** Descriptive statistics for temperature and temperature variability in summer and winter across the Southeastern USA, 2000–2013.

	Temperature (˚C)	Mean	Median	SD	Min	Max	Range	IQR	Q1	Q3
**Overall**	**Summer mean**	25.99	26.03	1.40	18.87	29.79	10.91	1.77	25.18	26.95
	**Winter mean**	9.42	8.65	4.57	−6.04	22.57	28.62	5.51	6.32	11.82
	**Summer SD**	1.66	1.63	0.37	0.76	3.15	2.39	0.53	1.39	1.93
	**Winter SD**	4.64	4.69	0.65	2.27	6.84	4.57	0.92	4.20	5.12
**Spatial**	**Summer mean**	25.99	26.03	1.24	20.28	28.77	8.49	1.48	25.32	26.79
	**Winter mean**	9.42	8.30	4.23	0.05	20.34	20.29	4.99	6.45	11.44
	**Summer SD**	1.66	1.71	0.27	1.08	2.33	1.25	0.48	1.41	1.88
	**Winter SD**	4.64	4.66	0.45	3.09	5.76	2.67	0.52	4.41	4.93
**Temporal**	**Summer mean**	0.00	−0.06	0.66	−2.17	1.98	4.15	0.85	−0.50	0.35
	**Winter mean**	0.00	0.42	1.73	−6.53	4.15	10.67	1.78	−0.66	1.13
	**Summer SD**	0.00	−0.04	0.24	−0.76	1.15	1.91	0.38	−0.19	0.19
	**Winter SD**	0.00	−0.05	0.47	−1.66	1.87	3.53	0.65	−0.33	0.32

**Table 3:** Percent increase in mortality (95% CI) for per 1 °C increase in seasonal mean temperature and temperature variability across the Southeastern USA, 2000–2013.

Temperature	Overall	*p*-value	Annual anomaly	*p*-value	Spatial contrast	*p*-value
**Summer mean**	2.46(2.33,2.59)	<0.001	0.96(0.72,1.19)	<0.001	4.49(4.24,4.75)	<0.001
**Winter mean**	−1.46(−1.50,−1.42)	<0.001	−1.27(−1.36,−1.17)	<0.001	−3.42(−3.51,−3.32)	<0.001
**Summer SD**	0.80(0.40,1.20)	<0.001	3.71(3.21,4.22)	<0.001	−18.46(−19.25,−17.67)	<0.001
**Winter SD**	0.41(0.22,0.60)	<0.001	0.59(0.37,0.81 )	<0.001	−3.49(−3.90,−3.08)	<0.001
