# Supplementary material for: Chronic effects of temperature on mortality in the Southeastern USA using satellite-based exposure metrics
Source: Sci Rep. 2016 Jul 20;6:30161. doi: 10.1038/srep30161 (PMC4951799; doi:10.1038/srep30161)
Supplement: Supplementary Information [file srep30161-s1.doc]

*Supplement of*

**Chronic effects of temperature on mortality in the Southeastern USA using satellite-based exposure metrics**

Liuhua Shi*, Pengfei Liu, Yan Wang, Antonella Zanobetti, Anna Kosheleva, Petros Koutrakis, Joel Schwartz

*Correspondence to:* [*lis678@mail.harvard.edu*](mailto:lis678@mail.harvard.edu)

**Table S1.** Correlation Matrix of variables studied


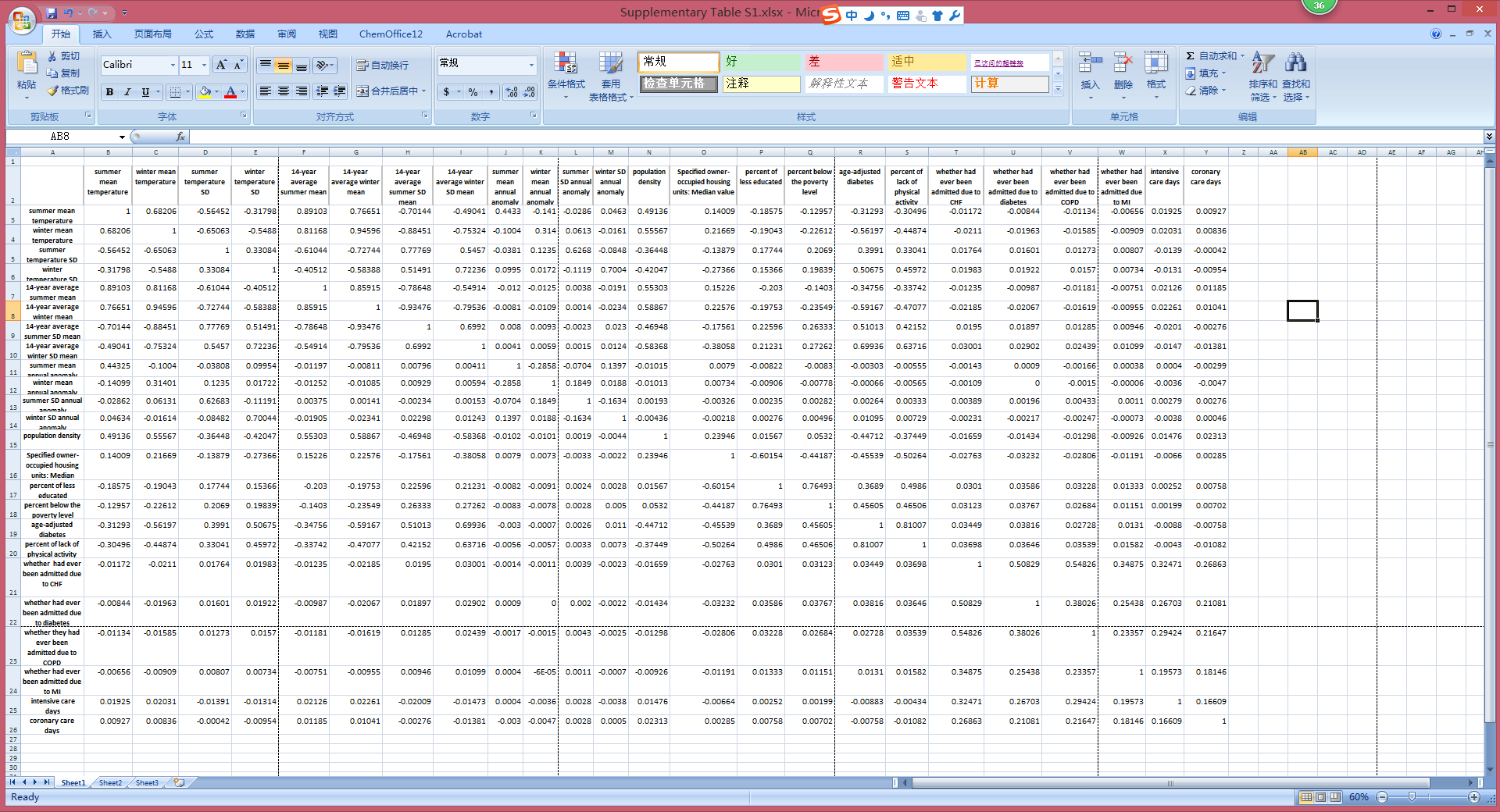


**Table S2.** Percent increase in mortality (95% CI) for per 1 ºC increases in seasonal mean temperature and temperature variability across the Southeastern USA in the sensitivity analyses

| **Sensitivity analyses** | **Temperature** | **Overall** | **Annual anomaly** | **Spatial contrast** |
| --- | --- | --- | --- | --- |
| **Cohort for**  **2000-2007** | **Summer mean** | 2.0(1.8,2.2) | 0.3(0.02,0.6) | 4.5(4.3,4.8) |
|  | **Winter mean** | -1.7(-1.8,-1.6) | -0.3(-0.5,-0.2) | -3.4(-3.5,-3.3) |
|  | **Summer SD** | 0.5(0.1,0.8) | 4.0(3.5,4.5) | -17.9(-18.7,-17.1) |
|  | **Winter SD** | 0.7(0.5,1.0) | 1.4(1.2,1.6) | -3.1(-3.5,-2.7) |
| **Control for Heat waves& cold waves** | **Summer mean** | 2.3(2.1,2.4) | 1.5(1.3,1.8) | 4.9(4.6,5.1) |
|  | **Winter mean** | -1.8(-1.9,-1.7) | -1.4(-1.5,-1.3) | -3.4(-3.5,-3.3) |
|  | **Summer SD** | 0.4(-0.01,0.8) | 4.5(4.0,5.1) | -17.6(-18.4,-16.8) |
|  | **Winter SD** | -0.3(-0.6,0.04) | 0.2(-0.1,0.5) | -3.2(-3.7,-2.8) |
